# Self-Healing Silicone Materials: Looking Back and Moving Forward

**DOI:** 10.3390/biomimetics8030286

**Published:** 2023-07-03

**Authors:** Konstantin V. Deriabin, Sofia S. Filippova, Regina M. Islamova

**Affiliations:** 1 Institute of Chemistry, St Petersburg State University, 7/9 Universitetskaya Emb., St. Petersburg 199034, Russia; deriabin.k@yahoo.com (K.V.D.); filippova.sonya@mail.ru (S.S.F.); 2 South Ural State University, Chelyabinsk 454080, Russia

**Keywords:** self-healing, silicone materials, reversible interactions, protective coatings, sensors, actuators, electroluminescent devices

## Abstract

This review is dedicated to self-healing silicone materials, which can partially or entirely restore their original characteristics after mechanical or electrical damage is caused to them, such as formed (micro)cracks, scratches, and cuts. The concept of self-healing materials originated from biomaterials (living tissues) capable of self-healing and regeneration of their functions (plants, human skin and bones, etc.). Silicones are ones of the most promising polymer matrixes to create self-healing materials. Self-healing silicones allow an increase of the service life and durability of materials and devices based on them. In this review, we provide a critical analysis of the current existing types of self-healing silicone materials and their functional properties, which can be used in biomedicine, optoelectronics, nanotechnology, additive manufacturing, soft robotics, skin-inspired electronics, protection of surfaces, etc.

## 1. Introduction

**Figure 1:**
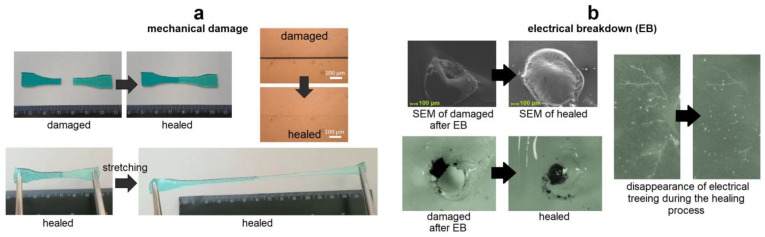
Demonstration of SH in SHSMs after mechanical damage [19,20] (**a**) and an electrical breakdown [9] (**b**). Reprinted with permission from [19]. Copyright 2021, American Chemical Society. Reprinted with permission from [9]. Copyright 2023, American Chemical Society. Copyright 2023, Elsevier.

The concept of self-healing materials originated from biomaterials (living tissues) capable of self-healing (SH) and regeneration of their functions at various levels: from deoxyribonucleic acid repair (micro level) to the healing of broken bones, blood vessels, plants (macro level), etc. Synthetic polymer materials are the most promising to create materials with a SH ability [1,2]. SH polymer materials can partially or entirely restore their original characteristics after mechanical damage (for example, formed (micro)cracks, scratches, cuts and ruptures [3,4,5]) or electrical damage (electrical breakdown, cracks and treeing [6,7,8,9]) is caused to them (Figure 1). The guiding principles for creating those materials can be found in the SH behavior for various biological systems [1,2], which includes a triggered actuation, transport of chemical agents and/or diffusion of polymer chains to the damage, and chemical repair process dependent on healing mechanism [1].

SH can be triggered by damage (autonomous) or external stimulus (non-autonomous) [6,10]. The SH of non-autonomous materials occurs via external stimuli, such as high temperatures or light. In the case of SH at high temperatures, the behavior of polymers is similar to thermoplastics (reversible healing) and thermosetting plastics (irreversible healing). Some authors [11] classify thermoplastics and thermosetting plastics as non-autonomous SH materials. However, this aspect is still debatable. Autonomous materials do not require external stimuli for their SH, and the damage itself is the stimulus to initiate the healing process [12].

The SH polymers are in high demand due to the increased durability and service life of materials and devices based on them. They can be applied to create SH coatings, highly stretchable strain sensors, electronic skins, actuators and artificial muscles, which are highly desirable for innovations in soft robotics, medicine, 3D printing, optoelectronics, etc. [7,11,13,14,15,16,17,18].

One of the most promising matrices among other elastomers [3] and hydrogels [21] to create SH materials is polydimethylsiloxane (PDMS) and its derivatives [3,6,18]. PDMSs exhibit high flexibility, stretchability, bioinertness, hydrophobicity, high chain mobility, high thermal stability, frost resistance, and a low glass transition temperature (−123 °C) [22,23,24]. PDMS is also resistant to UV radiation and ozone [6,23,25]. The high mobility of functionalized PDMS chains provides the SH properties of silicone materials, which results in silicone materials belonging to the extremely high diffusion of polymer chains between the damaged interfaces [23,25].

There are a number of reviews on PDMSs [18,22,23,24,25,26,27,28] and self-healing silicone materials (SHSMs) [3,6,18] in which SH mechanisms are preferably described. However, over the past five years, there was a revolutionary step in the development of functional SHSMs aimed at expanding practical areas of their application. Therefore, the main purpose of this review is a brief analysis of previously studied SHSMs (“looking back”), and those developed over the past 5 years, especially from the point of view of the use of functional SHSMs (“moving forward”).

## 2. Self-Healing Silicone Materials: Looking Back

In 2008, Cordier P. et al. pioneered the development of autonomous elastomers that can self-heal at room temperature (RT) via reversible hydrogen bonds [29]. The reversible chemical interactions used in such SH materials are classified as covalent and non-covalent bonds [3,6,10,14,16,30,31,32,33,34].

**Figure 2:**
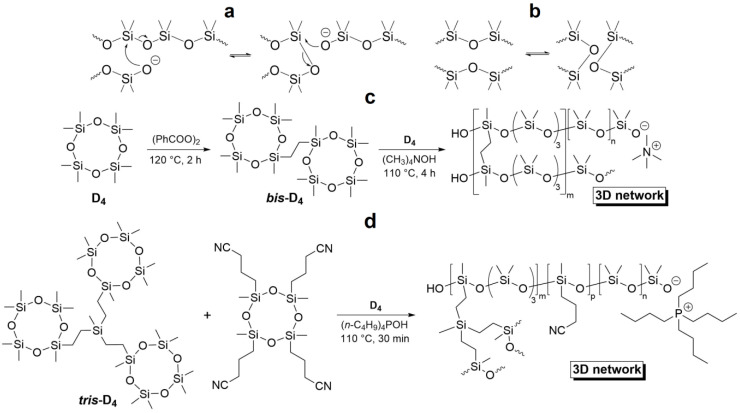
Siloxane equilibrium anionic-type mechanism (**a**), its general form of chain rearrangement (**b**) [35,36], and synthetic routes of SH polymer networks based on D_4_ and *bis-*D_4_ [35,36] (**c**) and *tris-*D_4_ [8] (**d**). Ph—phenyl group, **D_4_**—octamethylcycloteterasiloxane.

The discovery of the siloxane equilibrium facilitates the reversibility of the silicone cross-linking that opens up an opportunity to create SHSMs [8,35,36,37]. Thus, incorporation of anionic fragments into the three-dimensional (3D) polymer network by anionic ring-opening copolymerization of mono- (D_4_) and bicyclic (*bis*-D_4_) or tricyclic (*tris*-D_4_) oligosiloxanes provides a stable, dynamic restructuring of the silicone rubber’s structure (Figure 2) [8,35,36].

During these synthetic routes, the changes in the concentration of cross-linkers and initiators allowed for controlling of the cross-link density, tensile strength (*σ*), elongation at break (*ε*) and SH properties (self-healing efficiency, *η*). The optimal choice of the parameters allows materials to recover from damage in up to just a few seconds at RT. It should be noted [8,35,36] that the obtained “living” reactive anionic SHSMs are air-stable (are stable to water, oxygen, and CO_2_).

### 2.1. SHSMs Based on Covalent Interactions

**Figure 3:**
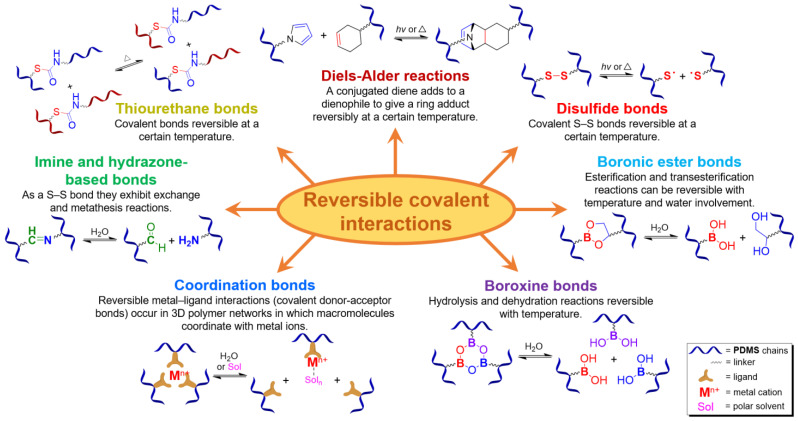
Some typical covalent intrinsic SH mechanisms used in known healable silicone materials. Δ—heating.

Figure 3 presents the SHSMs based on covalent interactions such as reversible Diels–Alder interactions [38,39,40,41,42,43,44,45,46], disulfide [47,48,49], boronic ester [50], boroxine [51,52], imine [53,54,55,56,57], hydrazine-based [58], thiourethane [59], hindered urea bonds [60], and metal–ligand interactions [19,61,62,63,64,65,66,67,68,69,70,71,72,73,74,75,76,77,78,79,80]. Some examples of covalent SHSMs are shown in Table 1.

#### 2.1.1. Self-Healing by the Diels–Alder Cycloaddition

The Diels–Alder cycloaddition reaction is one of the first and most commonly used reversible reactions to obtain SH polysiloxanes [38,39,40,41,42,43,44,45,46], and occurs at high temperatures (>50 °C) without a catalyst (Figure 3). The well-known SHSMs are based on the maleimide and furan groups. For instance, Nasresfahani A. and Zelisko P.M. proposed [43] the cross-linking of siloxane chains with pendant maleimide groups using a furan-functionalized polyhedral silsesquioxane [12].

In 2016, a method to prepare a SH polymer film via a thermally reversible interaction between a furan-modified polyurethane and a maleimide-containing hyperbranched polysiloxane was investigated by Fu G. et al. [39]. Modified carbon nanotubes were incorporated to the silicone matrix to obtain an electrostatic-dissipative SHSM with *η* = 93% after healing at 130 °C for 5 min. The resulting films have been reported [39] to have high opportunities for applications in the aerospace industry, electronics and other related fields [12].

Gou Z. et al. [40] developed a simpler method to prepare SHSMs via a thiol-ene click reaction of furan-functionalized tetravinyltetramethylcyclotetrasiloxane with bismaleimide. The obtained material maintains a strong UV-induced photoluminescence, and is of great potential for use as a glass binder [12].

A year later (2017), SH ladder structures based on polysilsesquioxanes were obtained [41,42] by utilizing pyrrolyl and cyclohex-3-enyl substituents as diene and dienophile, respectively. The presence of acrylic or epoxy groups in the material’s structure made it possible to achieve a higher cross-linking degree, thereby facilitating the convergence of polymer chains and results in highly efficient SH. According to the bibliography [41,42], the obtained materials have high thermal stability (>400 °C), improved tensile characteristics (Young’s modulus > 9 GPa), and a high optical transparency (up to 95%) [12].

Many SH silicone nanocomposites were prepared based on the Diels–Alder reaction between maleimide-modified polysiloxanes and furan-modified graphene [45,46]. Such materials exhibit *σ* = 0.25–1.09 MPa and *η* > 90%. It was noted [45,46] that these SHSMs have great potential for use in SH pressure sensors with high sensitivity (0.765 kPa^−1^) [12].

#### 2.1.2. Self-Healing by Imine and Hydrazone-Based Bonds

The introduction of imine bonds into a 3D silicone network cause non-autonomous SH properties of SHSMs at RT induced by water [53,54,55,56,57]. Zhang B. et al. [53] synthesized a PDMS elastomer cross-linked by imine bonds (Figure 3), which is characterized by acceptable optical transmission (80%), high *ε* ≈ 700%, and good SH properties (*η* = 53 and 100% after SH during 1 min and 1 h, respectively). Although SH occurs in water, it happens even at temperatures below −20 °C, which makes it suitable for optoelectronic and biotechnology applications [12].

In 2023, Lee J.M. et al. [54] used reversible imine bonding of aminopropyl-terminated PDMS and benzene-1,3,5-tricarboxaldehyde to fabricate corrosion-resistant coatings with SH ability. Both unscratched and healed coatings maintained outstanding corrosion inhibition efficiencies of up to 99%. In the last few years, some imine-based functional SHSMs have been prepared by some other authors [12,55,56,57].

**Figure 4:**
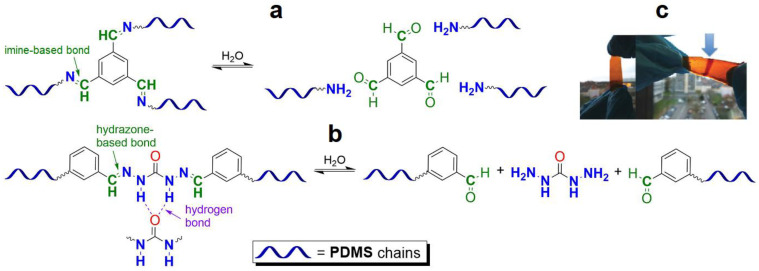
SH mechanisms in SHSMs based on imine [53] (**a**) and hydrazone bond [58] (**b**), as well as a demonstration of its SH [58] (**c**). Copyright 2013, John Wiley and Sons.

Similar hydrazone-based bonds are formed by the reaction of an aldehyde group with a hydrazide. The cleavage and formation of a hydrazine-based bonds are reversible and induced by an acid catalyst. Roy N. et al. [58] suggested using benzaldehyde-terminated PDMS for condensation with carbohydrazone to obtain an SHSM (Figure 4). In such systems, SH occurs at RT along with reversible hydrogen bonding, due to the action of acid catalysts (pentadecafluorooctanoic and adipic acid) incorporated into the silicone matrix. According to the ref. [58], the *η* achieves 100% when the damaged material is stored for more than 4 h [12].

#### 2.1.3. Self-Healing by Disulfide Bonds

The dynamic disulfide bonds dissociate into free radicals with an increase in temperature, and associate back when temperature declines. This fact allows for using of disulfide bonds as reversible cross-links to create non-autonomous [47,48] and autonomous [49] SHSMs (Figure 3). Wu X. et al. [48] obtained copolymers of PDMS and polyurethane by introducing a low-molecular-weight aliphatic disulfide. The synthesized materials exhibit high thermal stability (>300 °C), *ε* up to 1200%, and non-autonomous SH properties at 60–120 °C (*η* = 97%) [12].

In 2018, Lv C. et al. [47] reported a superstretchable PDMS-based SHSM capable of SH by dynamic S–S and imine bonds at RT, with good reproducibility (complete SH in 4 h) and *ε* = 2200%. Thus, the elastomer can be processed many times without reducing its mechanical properties, due to the presence of two reversible cross-links in the 3D polymer network [12].

In 2023, hierarchical covalent cross-linked networks and reversible bonds for flexible electronics were obtained by Zhang T. et al. [49]. The materials have *σ* = 0.87 MPa, *ε* = 410%, and heal at RT for 12 h by reaching *η* = 83% [12].

#### 2.1.4. Self-Healing by Boronic Ester and Boroxine Bonds

The boronic ester bonds are also used to create non-autonomous SHSMs, which heal via reversible hydrolysis and esterification in the presence of water. Thus, Zuo Y. et al. [50] introduced boric acid and glycol fragments into PDMS by hydrothiolation, developing SH silicone rubbers with dynamic boron-based cross-links (Figure 3). It was reported [50] that such materials achieved a *η* of up to 70% after 30 min under the influence of air moisture. For example, if a small amount of water is added to such SHSMs, the time of SH can be <10 min [12].

Lai J.-C. et al. [51] obtained a silicone rubber based on dynamic boroxine cross-linking (Figure 3). Initially, the SHSM is solid (*σ* = 10 MPa), rigid (Young’s modulus reaches 182 MPa) and withstands a load more than 450 times its weight. According to the ref. [51], SH is initiated by water or the addition of a Lewis base. In 2022, Liang H. et al. [52] obtained similar SH polymer networks based on boroxine cross-links with good SH ability (*η* = 98% after 4 h of water immersion), anti-icing performance, and superhydrophobic properties [12].

#### 2.1.5. Self-Healing by Thiourethane Bonds

In 2023, Qian Y. et al. [59] used a new type of reversible covalent cross-links to obtain non-autonomous SHSM—thiourethane bonds (Figure 3). In this report, a series of waterborne poly(thiourethane-urethane)s with dynamic thiourethane cross-links were prepared by click reactions. The materials exhibit non-autonomous SH at >90 °C (*η* = 69% after hot pressing at 140 °C). The authors noted [59] that incorporation of isobornyl acrylate increases the *σ* value from 4.70 to 12.56 MPa, which was approximately three times that of the original material.

#### 2.1.6. Self-Healing by Urea Bonds

**Figure 5:**
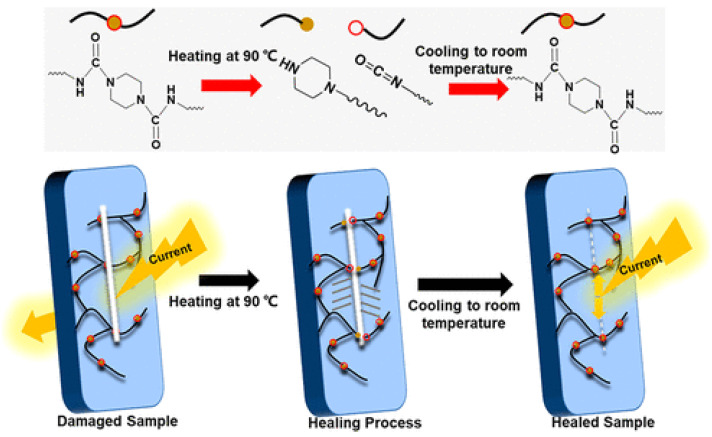
The structure of SH copolysiloxane, based on dynamically hindered urea bonds [60]. Reprinted with permission from [60]. Copyright 2021, American Chemical Society.

Dynamically hindered urea bonds were utilized [60] to create a smart insulating material with non-autonomous SH ability for powered and electronic devices. According to the ref. [60], such SHSMs were constructed by isocyanate–piperazine-based dynamic bonds with a cross-linking degree adjusted by glycerol. It caused not only healing the cut-damaged feature of the silicone, but also enabled dielectric property recovery after electrical breakdown (Figure 5). The obtained materials exhibit a *η* of above 95% and a recycling efficiency above 90%, based on insulation performance (after healing at 90 °C).

#### 2.1.7. Self-Healing Polymer-Metal Complexes Based on (Co)polysiloxanes

The metal–ligand coordination bonds are often dynamic, which makes it possible to prepare cross-linked SHSMs based on them (Figure 3) [3]. The metal–ligand coordination causes the formation of donor–acceptor bonds between a metal cationic center and donor non-metallic atoms of a ligand with an electron pair. Since a covalent bond is formed by two atoms sharing a pair of electrons, the metal–ligand coordination bonds belong to the category of covalent interactions. Thus, a coordinate bond (also called a dative covalent bond) is a covalent bond (a shared pair of electrons), in which both electrons come from the same atom [79,81,82].

The conditions required for the formation of coordination bonds between a copolysiloxane ligand and metal ion are relatively mild, which causes coordination cross-linking of polymer chains and the obtaining of polymer-metal complexes (PMCs). One of the important proses of this approach is that the mechanical and SH material’s properties can be relatively easily controlled by changing (i) the structure of the copolysiloxane ligand via changing the type of ligand-forming fragments (donor atoms—O, N, etc., carboxylate, mono-, bi-, terpyridine fragments, etc.), (ii) the molecular weight of the copolysiloxane ligand or PDMS chain length, (iii) the metal–polymer ligand ratio, and (iv) the metal ion and counterion content [12].

**Figure 6:**
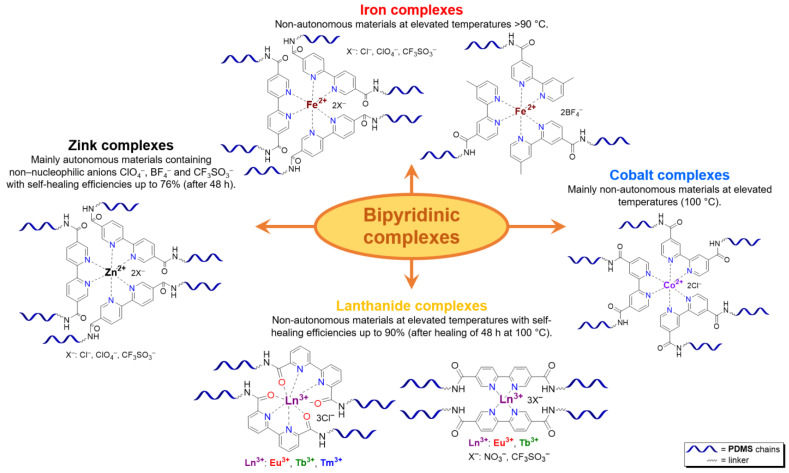
Some PMCs based on bipyridine-containing PDMS [19,61,62,63,64,65,80].

**Bipyridinic PMCs.** One of the first prepared PMCs based on PDMS and a metal cation was bipyridine-containing SHSMs. In 2016, the scientific group of Bao Z. [61] firstly obtained a SH dielectric elastomer by metal–polymer ligand coordination acting as cross-links of PDMS chains (Figure 6). The polymer ligand was poly(2,2′-bipyridine-5,5′-dicarboxamide-*co*-PDMS), and metal salts are Fe^2+^ and Zn^2+^ with different anions (BF_4_^−^, ClO_4_^−^, CF_3_SO_3_^−^). The flexibility of PDMS chains provides a sufficiently high mobility in the 3D polymer network structure to enable reversible coordination of metal ions and bipyridine at RT. The kinetically labile coordination between Zn^2+^ and bipyridine allows the silicone rubber to SH rapidly and autonomously at RT. Thus, the *η* of Zn(CF_3_SO_3_)_2_-PMC is ca. 76%, while the *η* of Zn(ClO_4_)_2_-PMC and ZnCl_2_-PMC are 55 and 21%, respectively. Compared to the labile Zn^2+^-based PMCs, Fe^2+^-based bipyridinic PMCs demonstrated non-autonomous SH upon heating to 90 °C for several hours, due to the kinetically inert coordination of Fe^2+^–N_bipyridyl_ at RT, and the overall contribution of the higher mobility of PDMS chains and the more labile coordination of Fe^2+^–N_bipyridyl_ at elevated temperatures [61]. The described obtained SHSMs were used in flexible electronics, for example, to produce artificial skin (“electronic skins”) and artificial muscles [12].

Three years later, in 2017, similar redox-active PMCs based on bipyridine-containing PDMS (with a number-average molecular weight *M_n_* = 3300–50,000) and Fe^2+^ were synthesized (Figure 6) [62]. In 2017 and 2022, non-autonomous bipyridine-based PMCs [65,80] were prepared. These Eu^3+^-, Tb^3+^-, and Tm^3+^-containing PMCs (Figure 6) showed a relatively high *σ* (1.5 MPa), *ε* ≈ 185%), and exhibited non-autonomous SH properties (*η* ca. 90% on heating at 100 °C after 2 days) [12].

**Figure 7:**
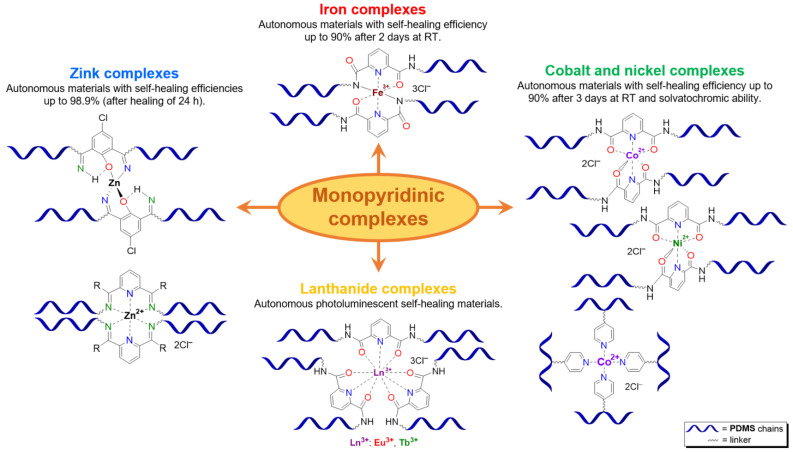
Some PMCs based on monopyridine-containing PDMS [63,66,67,68,69,70].

**Monopyridinic PMCs.** In 2016, Bao Z. et al. [66] partially completed the task of obtaining materials that have the properties of biological muscles—superstretchability and SH. PMCs, based on the coordination of poly(pyridine-2,6-dicarboxamide-*co*-PDMS) (Py-PDMS) and FeCl_3_·6H_2_O, exhibit a record high *ε* (1000–10,000%), high dielectric strength, and autonomous SH properties (*η* = 92% after healing for 48 h) at RT (Figure 7). It was reported [66] that the SH process occurs at low temperatures, down to −20 °C (*η* = 68% after healing for 72 h). The authors suggested [66] applying these Fe^3+^-PMCs to fabricate artificial muscles and electronic skins [12].

As in the case of the above-considered Zn^2+^-based bipyridinic PMCs, the high lability of Zn^2+^-containing monopyridine-based PMCs has allowed the synthesis of other elastic PMCs based on Zn^2+^ (*ε* up to 400%, *σ* = 0.05–3.5 MPa) with *η* = 100% after SH at RT (Figure 7) [12,67,68].

In 2015 and 2017, Jia X.-Y. and Liu L. et al. synthesized [63,69] an autonomous PMC with Co^2+^ in their structure (Figure 7). As distinguished from Fe^3+^- and Zn^2+^-PMC [66,67,68], Co^2+^-PMC exhibit solvatochromic properties—a color change on contact with polar solvents (alcohols and acetonitrile). Some years later, in 2020–2021, Deriabin K. et al. [19,64] obtained an autonomous PMC of Py-PDMS with Co^2+^ and Ni^2+^ (Figure 7). Despite the fact that these PMCs had lower SH ability (self-healing efficiencies reaching 93% at RT after 72 h) and were less elastic, they were more durable and exhibited higher *σ* (up to 0.8–1.8 MPa) [12].

**Figure 8:**
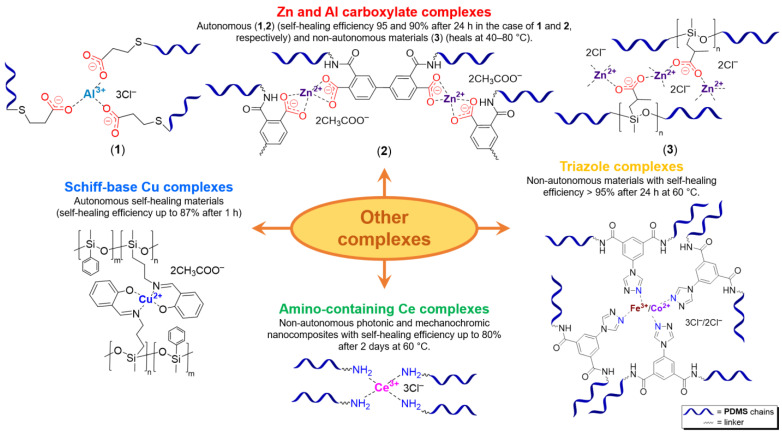
Some PMCs based on carboxylate-, amino-, triazole- and Schiff base-containing PDMS [71,72,73,74,75,77].

**Carboxylate-based PMCs.** Lai J.-C. et al. [71] developed rigid non-autonomous PMCs based on the labile interactions of Zn^2+^–carboxylate, which are sensitive to temperature. These SHSMs incorporated large amounts of coordination cross-links (50 mol.% of total polymer units) (Figure 8). It was shown [71] that *σ* values decline from 9 to 0.1 MPa with an increasing temperature from 25 to 70 °C. In accordance with these characteristics, these SHSMs exhibit rapid SH at elevated temperatures, making them suitable for additive technology and orthopedic applications [12].

In 2022, Au-Duong A.-N. et al. [72] obtained a material with polyamic acid and Zn^2+^ in its structure, exhibiting high extensibility, toughness and spontaneous autonomous SH. The described SHSMs are transparent, maintain a good *σ* of 0.27 MPa, and have high stretchability (*ε* = 360%) [12].

Subsequently, autonomous PMCs based on metal–carboxylate interactions with Al^3+^ metal centers were synthesized (Figure 8) [73]. The tensile (*σ* = 0.1–1.1 MPa, *ε* = 10–140%) and SH characteristics of Al^3+^-based PMCs can be controlled by changing the polymerization degree of cyclic oligosiloxanes, grafting density of carboxyl, and concentration of Al^3+^ [12].

**Triazole-, amino- and Schiff base-based PMCs.** PMCs based on autonomous imidazole-Zn^2+^- [76] and non-autonomous triazole-containing PDMS [77] are known. In 2016, Jia X.-Y. et al. [77], by incorporating dynamic Fe^3+^ and Co^2+^-triazole coordination bonds into PDMS chains, prepared a highly elastic SHSMs (*ε* = 3400%) (Figure 8). The polymers maintain non-autonomous thermal SH properties (*η* = 90% after healing at 60 °C for 20 h).

In 2017, Yu D. et al. [74] used the metal center to Cu^2+^ to obtain PMCs by the reaction between poly((3-aminopropyl)methylsiloxane-*co*-methylphenylsiloxane)s and salicylaldehyde-forming imine groups, then complexation of the obtained ligand with Cu(CH_3_COO)_2_ (Figure 8). The dynamic character of the metal–polymer ligand interactions of copolysiloxanes with pendant Schiff base-groups with Cu^2+^ in a SHSM caused a high ability for autonomous SH at 30 °C (*η* = 87% after healing for 1 h) [12].

In 2018, Tan H. et al. [75] utilized the simplest method to prepare a SHSM on the basis of the complexation reaction between CeCl_3_ and an amino-terminated PDMS (Figure 8). The obtained nanocomposites with SiO_2_ and carbon black exhibited non-autonomous SH properties in combination with photonic, angle-independent color, and mechanochromic characteristics [12].

Thus, PMCs incorporate multidentate ligands, including nitrogen-containing aromatic rings [19,61,62,63,64,65,66,67,68,69,74,83], amines [75], imidazole [76], triazole [77], or carboxylates [71,73]. Mono- [63,66,67,68,69], bipyridine fragments [19,61,62,63,64,65], and Schiff bases [74] are used in most ligands, and can improve the mechanical properties of materials. Iron [61,62,66,77,83], cobalt [19,63,69,77,84], zinc [61,67,68,71,76,83], aluminum [73,85], nickel [64], lanthanides [65,75,86], copper [74,83], and platinum [87] are mainly utilized as metal centers. The monopyridine-containing PMCs mostly maintain autonomous SH ability, compared to bipyridine-containing ones [12].

In view of the foregoing, SHSMs based on covalent interactions exhibit predominantly non-autonomous SH properties and require external action, which is associated with stronger bonds in their structure and increased dissociation energy. For example, (i) heating (in the case of Diels–Alder interactions [38,39,40,41,42,43,44,45,46], disulfide [47,48,49], thiourethane [59]), (ii) UV, and (iii) the additional reaction agent (the addition of water in the case of imine [53,54,55,56,57], boronic ester [50] and boroxine bonds [51,52]). This cannot be called an unambiguous advantage or disadvantage since, in some cases, SHSMs may be in demand if they self-heal only under special conditions. On the other hand, a number of autonomous SHSMs based on non-covalent interactions have been developed to date.

### 2.2. SHSMs Based on Non-Covalent Interactions

**Figure 9:**
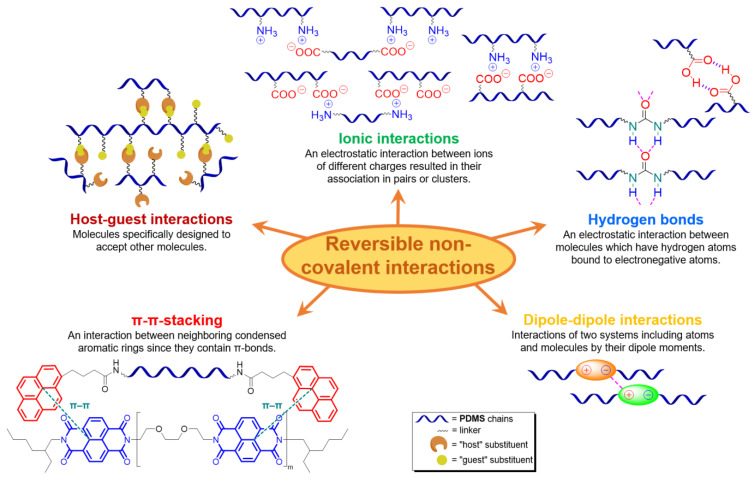
Typical non-covalent intrinsic SH mechanisms used in known healable silicone materials.

Along with SHSMs based on covalent reversible cross-links, there are elastomers with dynamic non-covalent interactions: hydrogen [88,89,90,91,92,93,94,95,96,97,98,99,100,101,102,103,104,105,106,107] and ionic bonding [108,109,110,111], π–π-stacking [87,112,113], metal–metal [87], and host–guest interactions [114] (Figure 9). Some examples of non-covalent SHSMs are shown in Table 1.

#### 2.2.1. Self-Healing by Hydrogen Bonds

**Figure 10:**
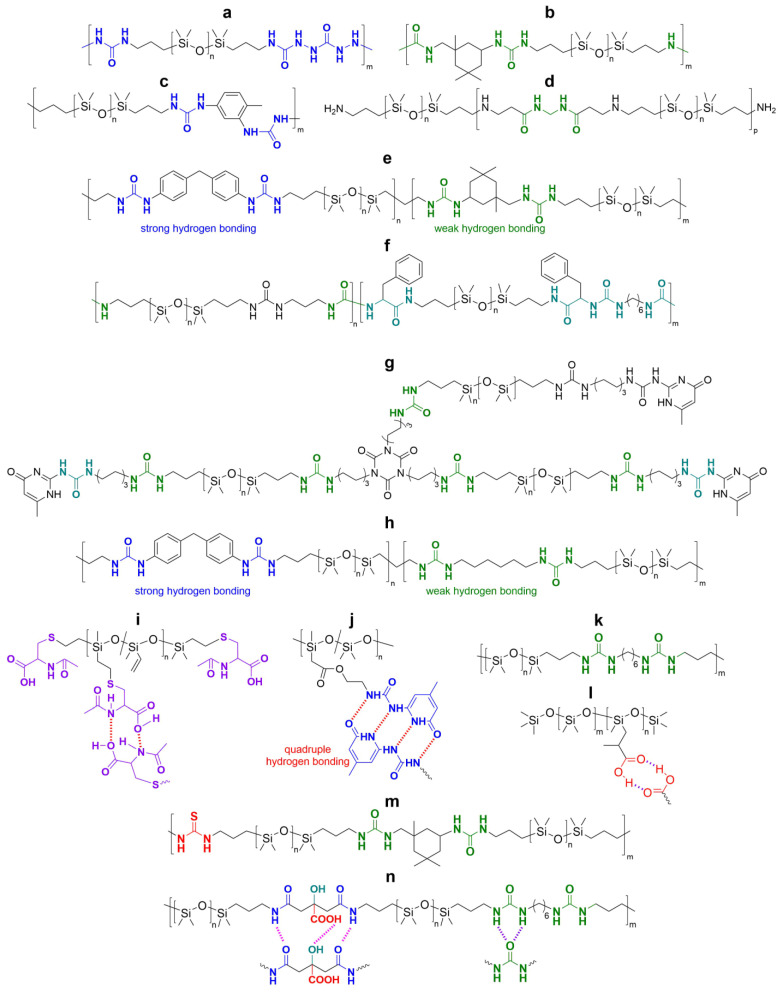
Some examples of hydrogen-bond-type SHSMs [89] (**a**), [91] (**b**), [90] (**c**), [98] (**d**), [92] (**e**), [93] (**f**), [94] (**g**), [99] (**h**), [100] (**i**), [101,102] (**j**), [103] (**k**), [107] (**l**), [104] (**m**), and [105] (**n**).

Hydrogen bonding is the most common reversible interaction used to form SHSMs [88,89,90,91,92,93,94,95,96,97,98,99,100,101,102,103,104,105,106,107] (Figure 10). The hydrogen bonds are chemical bonds between an electronegative atom (N, O, or F) and a hydrogen atom covalently bonded to another electronegative atom. The hydrogen bonds are characterized by their relatively low energy (an order of magnitude lower) in comparison with the covalent bonds discussed before. The hydrogen bonds occupy an intermediate position between chemical bonds and van der Waals forces. Since hydrogen bonds are intermolecular bonds, they cause the formation of dynamic cross-links between macromolecules, especially PDMSs containing polar groups. The hydrogen bonds are highly directional and can initiate self-assembly of polymer chains [12].

Zhang A. et al. [88] were the first to synthesize novel supramolecular SHSMs from a mixture of PDMS derivatives containing multiple COOH-groups, diethylenetriamine and urea. The obtained SHSMs have a low glass transition temperature of ca. −113 °C, rubber-like behavior, and autonomous SH properties at temperatures lower than RT [12].

In 2013, Roy N. et al. [89] prepared supramolecular networks with multiple hydrogen bonds by polycondensation of isocyanate-terminated PDMS with carbonylhydrazine (Figure 10a). This synthetic method can provide a wide range of materials with different flexibility, by proper selection of the bis-isocyanate component forming the polymer backbone. Such samples autonomously heal within a few hours. However, the polymers lose their SH properties if the cut samples are stored in the air for more than 10 min [12].

In 2016, P. Baek et al. [90] obtained conductive composite materials based on a PDMS-urea block copolymers and poly(3-hexylthiophene). These SHSMs exhibited a mechanical *η* = 55%, and the electrical conductivity was restored by 82%. The authors noted [90] that simple mixing of PDMS-urea and poly(3-hexylthiophene) solutions is sufficient to obtain composite materials, which are suitable for the creation of flexible electrodes [90].

Similar to the Baek P. work [90], Liu C. et al. [91] obtained SH silicone coatings via polycondensation of bis-isocyanates and amino-terminated PDMS to protect marine vessels from biofouling (Figure 10b). The resulting antifouling-containing composites exhibited high *η* values (98–100%) and relatively good mechanical properties (*σ* = 0.81 MPa, *ε* of up to 550%) [12].

In 2018, the scientific group of Bao Z. et al. [92] synthesized SH copolysiloxane combining strong and weak hydrogen bonds via the reaction of amino-terminated PDMS with 4,4′-methylenebis(phenyl isocyanate) and isophorone diisocyanate (Figure 10e). The obtained polymer network has very high fracture energy (≈12,000 J∙m^−2^) and high *σ* values (1.5 MPa) and *ε* = 1200–3000%. The authors propose [92] the use of such SHSM as a rigid and elastic SH artificial skin for various electronic devices, opening new fields in soft robotics and skin prostheses [12].

In the last five years (2018–2023), various hydrogen-bonded SHSMs and composites based on them have been obtained [93,94,95,96,97,98,99,100,101,102,103,104,105,106,107]. These SHSMs are based on PDMS copolymers and urethanes, nitrogen-containing heterocyclic, and amine-containing moeties (Figure 10c–n). They lead to relatively fast autonomous SH properties within 5–120 min and have a wide range of mechanical properties (*σ* = 0.1–5.5 MPa) and *η* = 10–100%, which depend on the structure of the 3D polymer network. A notable achievement [97] was the synthesis of SHSMs by adding a “sliding” cross-linker (polirotaxanes) and hydrogen bonds into the polymer network, resulting in high values of *ε* (2800%), *σ* (1.05 MPa), and *η* (93% at 55 °C). This excellent extensibility is explained by the “sliding” of the cyclodextrins along the polysiloxane chains, and dynamic hydrogen bonding if deformation occurs [12].

#### 2.2.2. Self-Healing by Ionic Bonds

Ionic bonding can promote reversible cross-link formation in polysiloxanes mainly through interactions between amino groups and acid residues incorporated into the polymer structure [108,109,110,111]. In 2016, Madsen F.B. et al. reported [108] an autonomous SH dielectric elastomer consisting of an interpenetrating polymer PDMS network with a high dielectric constant, which cross-linked by proton exchange with an ion-containing polysiloxane between NH_2_– and COOH-groups (Figure 9). Ion-cross-linked polysiloxanes exhibit SH at RT after electrical breakdown or cleavage (up to 77% efficiency). The authors reported [108] that such SHSMs pave the way to improve the lifetime of dielectric elastomers and the ability to withstand millions of cycles under high voltage conditions, such as rupture and electrical breakdown, in contrast to conventional dielectric elastomers [12].

In 2019, Li Z. et al. [109] investigated a novel type of SHSM, which is cross-linked by irreversible covalent bonds and ionic interactions between Cl^−^/quartenized ammonia groups, as well as exhibited relatively good tensile properties (*σ* = 0.2–0.4 MPa, *ε* = 50–135%), SH (*η* ≈ 83% after being healed at RT for 12 h), and a high ionic conductivity (up to 1.19 mS∙cm^−1^ at 25 °C). It was reported [109] that ionogel shows good adhesion to various solid materials, and can retain its high ionic conductivity and SH even at temperatures < 0 °C. Such SHSMs are expected to be useful in the construction of flexible electronic devices, including sensors and supercapacitors, even at low temperatures (from −20 °C) [12].

In 2022, Boumezgane O. et al. [110] developed SH anticorrosive composite coatings via modification of a commercial epoxy coating by adding microcapsules composed of a poly(methyl methacrylate) shell and a core of ionic PDMS oligomers. The ionic-type cross-linking was also used to obtain other dielectric composites including elastomer generators [111].

#### 2.2.3. Self-Healing by π–π-Stacking

π–π-stacking usually occurs between aromatic rings due to overlapping *p*-orbitals in π-conjugated systems. This intermolecular interaction is dynamic, reversible and can be used in the synthesis of SHSMs [87,112,113]. In 2010, Burattini S. et al. [112] prepared SH copolymers via π–π-stacking between polyimide (with a deficiency of π-electrons) and pyrenyl-terminated PDMS (with an excess of π-electrons) for the first time (Figure 9). According to the ref. [112], as the temperature decreases, the π–π interactions cause the formation of new 3D polymer networks [12].

#### 2.2.4. Self-Healing by Intermolecular Metallophilic Interactions

**Figure 11:**
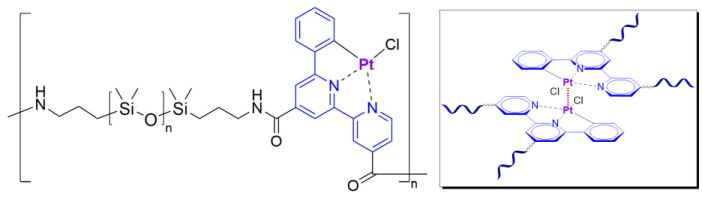
Structure of SH polymer based on intermolecular metallophilic Pt–Pt interactions [87].

In 2016, Mei J.-F. et al. [87] synthesized a SHSM based on intermolecular metallophilic Pt–Pt interactions by incorporating a cyclometallated Pt(II) complex and 6-phenyl-2,2′-bipyridyl into a PDMS backbone (Figure 11). The obtained elastic material can be stretched to more than 20 times its original length (*ε* > 2000%, *σ* = 0.38 MPa) and exhibits autonomous SH after damage at RT for 12 h. In this case, the SH of the SHSM is insensitive to changes in surface aging; and the SH ability rises with increasing temperature.

#### 2.2.5. Self-Healing by Host-Guest Interactions

**Figure 12:**
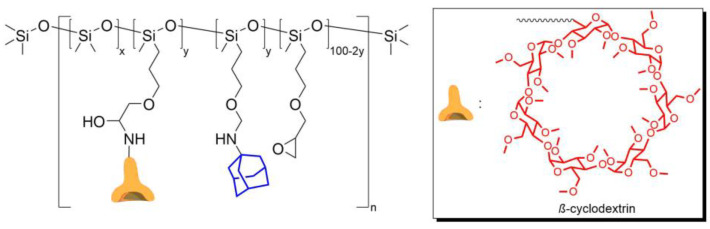
Structure of SHSM based on host-guest interactions [114].

In 2023, Daichi Y. et al. [114] obtained copolydimethylsiloxane with fragments of adamantane and beta-cyclodextrin methyl ether, the interaction between which formed the basis of reversible cross-linking (Figure 12). The paper notes that the resulting silicone material has a low *ε* (up to 76%), as well as a low *η* (30% at RT for 24 h).

**Table 1:** Tensile and SH properties of some non-composite SHSMs based on covalent and non-covalent dynamic bonds.

Type of Reversible Interaction	Simplified Structure of SHSM ^1^	Maximal Values			SH Conditions	Refs.
		*σ*, MPa	*ε*, %	*η*, % (Time of SH)		
Diels-Alder	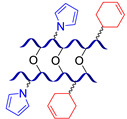	— ^2^	— ^2^	100% (5 min)	110 °C	[41,42]
Imine bonds	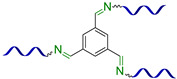	0.35	700	53% (1 min)	H_2_O, RT, down to −20 °C	[53]
Hydrazone bonds	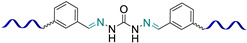	0.56	115	90% (—)	RT	[58]
Disulfide bonds	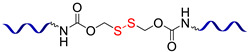	3.06	1200	97% (3 h)	60–120 °C	[48]
	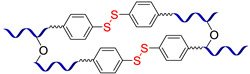	0.87	410	83 (12 h)	RT	[49]
Disulfide + imine bonds	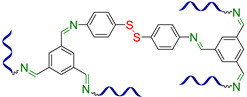	0.31	2200	95% (4 h)	60 °C	[47]
Boronic ester bonds	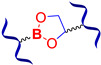	1.28	— ^2^	70% (30 min)	H_2_O, RT	[50]
Boroxine bonds	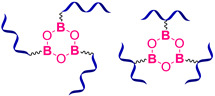	10	10	98% (4 h)	H_2_O, RT	[51,52]
Thiourethane bonds	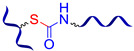	12.56	100	69 (12 h)	140 °C	[59]
Urea bonds	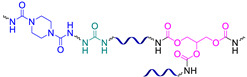	1.3	95	95 (12 h)	90 °C	[60]
Coordination bonds	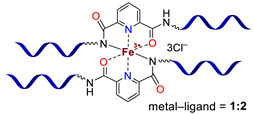	0.23	1860	92 (48 h)	RT, down to −20 °C	[66]
	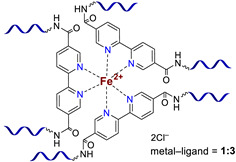	0.55	125	100	90 °C	[61]
	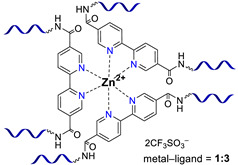	0.70	310	76 (48 h)	RT	[61]
	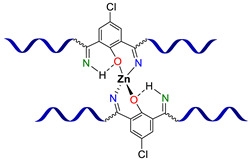	3.22	2400	77 (6 h)	RT	[68]
				99 (24 h)		
	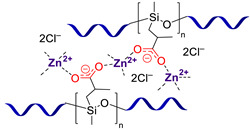	9.15	3.4	98 (4 h)	80 °C	[71]
Hydrogen bonds	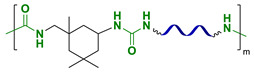	0.81	550	27 (4 h)	RT	[91]
				100 (4 h)	60 °C	
	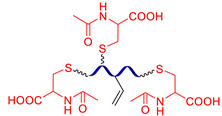	4.36	590	97 (24 h)	RT	[100]
	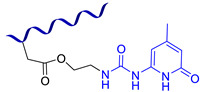	0.48	2077	85 (2 h)	60 °C	[102]
Ionic bonds	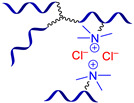	0.40	135	83 (12 h)	RT	[109]
π–π-stacking	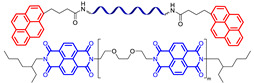	— ^2^	— ^2^	95	100 °C	[112]
Metallophilic interactions	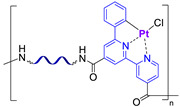	0.38	2000	100 (12 h)	RT	[87]

^1^ The symbols 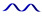 and 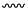 mean a siloxane (PDMS) chain and a linker, respectively; ^2^ the parameters have not been mentioned.

### 2.3. SHSMs Based on Interactions between Nanoparticles and Polymers

Nanocomposites with a certain range of properties are very often created based on SHSM with covalent (Diels–Alder reactions, coordination bonds, etc.) [38,115] and non-covalent types of reversible bonds (hydrogen bonds, etc.) [20,116,117] between nanoparticles (NPs) and polymer matrices in their structure.

**Figure 13:**
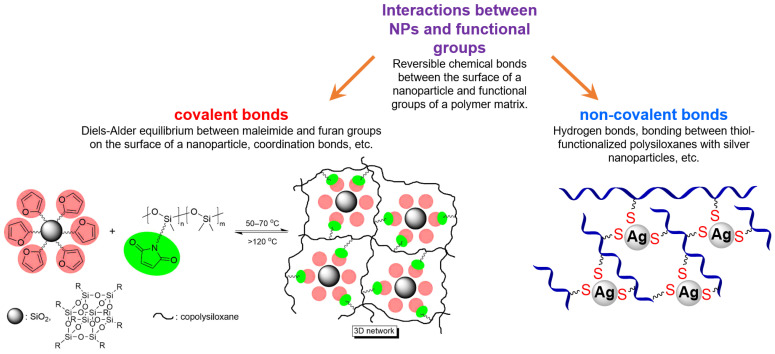
SH nanocomposites based on interactions between nanoparticles and polymers [12,20,38,43,115,116,117]. The red and green circles in the scheme mean furan and maleimide groups, respectively. Copyright 2015, Elsevier.

In 2015, Schäfer S. et al. [38] prepared a SH nanocomposite consisting of polysiloxane modified with a furan or maleimide group and SiO_2_ NPs functionalized with maleimide or furan (Figure 13) [12]. It was reported [38] that when a damaged sample is heat-treated, the rate of its SH will be low, but if CHCl_3_ is dropped on the damage or cut and then heated, the cut will disappear completely.

Jin K. et al. [117] obtained robust SH superhydrophobic surfaces by the design of healable superhydrophobic micro/nano rough surface. To obtain the SH coating, the silica-amino silicone oil (shell-core) particles (SiO_2_@NH_2_-PDMS) and PDMS were cross-linked by the formation of hydrogen bonds on the PA@PDA/AgNPs surface (Ag NPs on polydopamine-functionalized polyamide fabric). The interactions between Ag NPs and thiol groups are also used to create non-covalently cross-linked SHSMs. For instance, Martín R. et al. [116] have cross-linked thiol-modified polysiloxanes with Ag NPs (Figure 13). The obtained composites maintain relatively good mechanical properties (*σ* = 0.35 MPa, *ε* = 60–80%) and high autonomous SH properties within 24 h at RT [12].

Non-covalent bonds are weaker interactions compared to covalent ones that mainly lead to autonomous SH (especially hydrogen and ionic bonds). However, it is necessary to form a sufficiently large number of weak non-covalent bonds to form stable, mechanically strong, and durable SH polymer structures. In this regard, SHSMs with combined interaction are being developed.

### 2.4. SHSMs Based on Combination of Interactions

**Figure 14:**
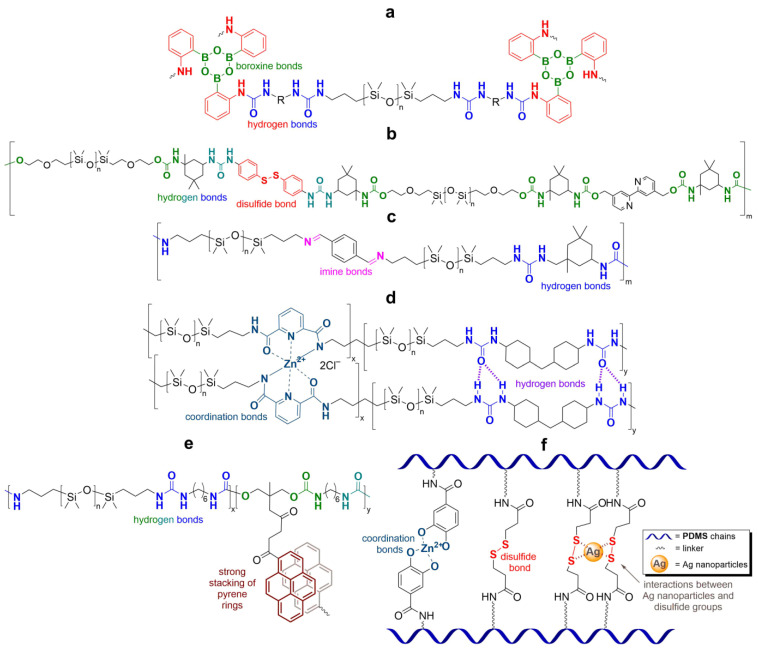
Formulae of some SHSM with double and triple combinations of reversible cross-links: boroxine and hydrogen bonds [119] (**a**), disulfide and hydrogen bonds [121] (**b**), imine and hydrogen bonds [125] (**c**), coordination and hydrogen bonds [130] (**d**), π–π-stacking and hydrogen bonds [136] (**e**), coordination, disulfide bonds and interactions between Ag nanoparticles and disulfide groups [141] (**f**).

Current approaches to create SHSMs often use a combination of different interactions (Figure 14); for instance, combinations of dynamic covalent and non-covalent bonds: (1) boronic and hydrogen bonds [118], (2) boroxine and hydrogen bonds [119], (3) disulfide and hydrogen bonds [120,121,122,123], (4) imine and hydrogen bonds [124,125,126,127], (5) phenol carbamate and hydrogen bonds [128], (6) coordination and hydrogen bonds [129,130,131,132,133], and (7) metal–ligand interactions with various metals [70].

In the last few years, utilizing double combinations of non-covalent bonds is also frequent, in the form of (1) ionic and hydrogen bonds [134,135] and (2) π–π-stacking and hydrogen bonds [136]. In the case of double-cross-linked SHSMs using reversible imine and coordination bonds [83], the mechanical properties of elastomers can be tuned by adjusting the type and content of metal ions.

Interactions between NPs and functional groups were also used in combination with hydrogen bonds [137,138,139], vinylogous urethane bonds [140], and coordination metal–ligand bonds [115].

In addition, compared to single and some dual cross-linked elastomers, the use of triple combinations of disulfide, coordination and hydrogen bonds [141,142], and of π–π-stacking, disulfide and hydrogen bonds [143] leads to obtaining SHSMs with notably enhanced mechanical properties (*σ* is up to 3.5 MPa and *ε* is up to 1800%). Thus, the mixing of different interactions allows for giving the material a variety of properties, including faster healing or improved mechanical characteristics.

## 3. Applications of Self-Healing Silicone Materials: Moving Forward

**Figure 15:**
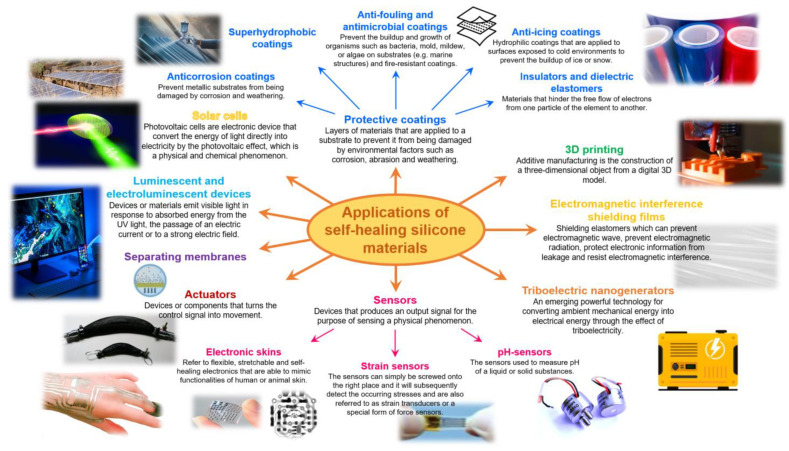
Main typical types of applications for known SHSMs.

In the past five years, SHSMs have become one of the most promising field of investigation in materials science, which is confirmed by the huge publication activity. In general, silicone rubbers with SH ability have broad applications in nanotechnology, optoelectronics, biomedicine, soft robotics and human activity [11,13,14,15,16,17]. Various protective coatings with a long service life [9,26,27,53,54,110,120,125,139,144,145], electromagnetic shielding films [57,99], triboelectric nanogenerators [126,130], sensors and skin-inspired electronics [49,101,118,124,127,129,131,134,136,141,143,144,146,147,148,149], actuators and artificial muscles [8,44,66,107,118,126,147,148,150], separating membranes [151], liquid crystals [152], and flexible and stretchable luminescent and electroluminescent devices [18,55,56,65,78,83,105,132,146], as well as solar cells [113,137,138], are created based on functional SHSMs depending on their additional properties, e.g., thermal and cold resistance, redox-activity, luminescence, high dielectric properties or electrical conductivity, etc. (Figure 15). Moreover, flexible, twistable and stretchable self-repairing silicone materials can be used in additive manufacturing and for various applications, such as in orthopedic immobilization, conductive composites/adhesives, and 3D printing [71,153].

### 3.1. Recent Developments in Protective Coatings

Industry utilization of protective coatings are many, and straddle the automotive, aerospace, marine, building, and fuel industries. For instance, in the automotive and aerospace industries, protective coatings are used to prevent corrosion and weathering on car or aircraft surfaces and components. There are also several other types of protective coatings, including inhibitive coatings, which contain chemical substances that prevent corrosion, weathering, icing, fouling, and combination coatings, which are made up of two or more coatings. SHSMs create more potential to increase the service life and reliability of protective coatings. Thus, SH silicone rubbers based on the covalent and non-covalent reversible interactions (imine, disulfide, boroxine, carbamate, hydrogen, ionic and coordination bonds) were utilized to prepare various types of protection coatings, including anticorrosion films [53,54,110,120,125,139,144], antifouling and antimicrobial coatings [20,100,104,106,120,145], insulators [44,60,154], films with SH ability after electrical breakdown [7,8,9,60,61,108], anti-icing [121], and superhydrophobic coatings [26,52,119,155]. Thus, there are no specific formulas related to protective coatings, as the composition and properties of the coating will vary depending on the specific application.

#### 3.1.1. Self-Healing Anticorrosion Coatings

**Figure 16:**
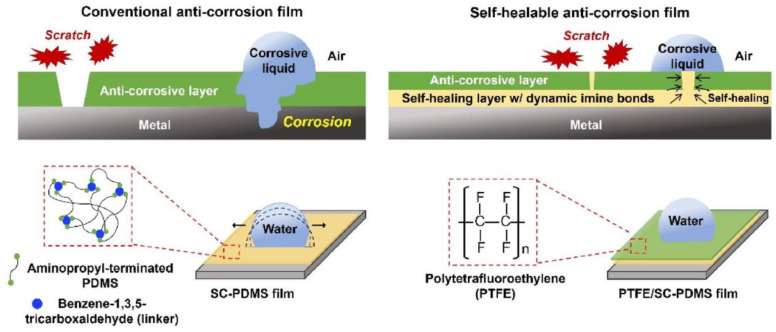
Difference between conventional (**left**) and long-term SH anticorrosion (**right**) films [54]. Copyright 2023, Elsevier.

One of the most recent developments in anticorrosion protective coatings was ref. [54] from Lee J.M. et al. In this study, a combination of SH and fluorinated polymers was used to develop an autonomous SH hydrophobic coating with excellent corrosion resistance that allows protecting metal surfaces from scratches by a reproducible damage-healing property. The authors used a Schiff-base-linkage-based PDMS (SC-PDMS) with the dynamic imine bonds as a primary protection layer against corrosive media. The thin film of polytetrafluoroethylene at the SC-PDMS surface quenches the reaction of the imine bond with water and improves the long-term scratch-free corrosive resistance (Figure 16).

**Figure 17:**
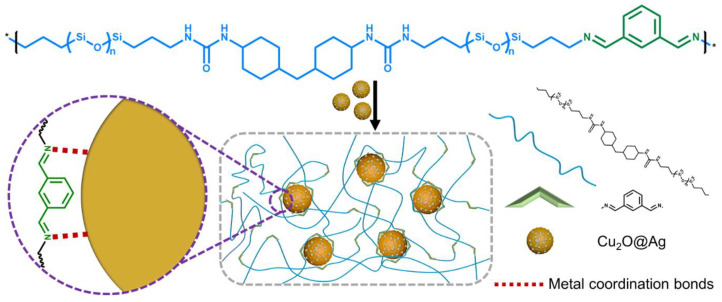
Synthesis of PDMS-Cu_2_O@Ag anticorrosion protective film [139]. Copyright 2022, Elsevier.

A combination of dynamic imine and multiple hydrogen bonds allowed Mo P. et al. in 2022 [125] to create anticorrosion coatings with additional self-cleaning performance. In 2022, Wang T. et al. used [139] similar hydrogen bonds, combined with imine and noticeable coordination bonds between nanofillers (Cu_2_O@Ag), and nitrogen atoms of a PDMS matrix, to fabricate multiple cyclic and long-term SH silicone coating, which is reinforced by Cu_2_O@Ag (Figure 17). At the same time, Ji X. et al. [144] obtained anticorrosion coating with wide pH-responsive and SH performance based on core–shell nanofiber containers. The fiber-PDMS coating exhibits *η* = 96 and 97% in alkaline and acidic solutions, respectively [144]. The SH epoxy coatings with microencapsulated ionic PDMS oligomers were also used by Oussama B. et al. [110] for corrosion protection based on acid-base ionic interactions.

Some recent investigations led to the synthesis of anticorrosion films with specific antifouling and antimicrobial properties [103,120]. The first polymer coating [103] featuring multifaceted functionalities was prepared by facilely brush-coating isocyanate-modified PDMS on various substrates, in which the adjacent polymer chains are physically cross-linked by the hydrogen bonds between the urea moieties. The authors noticed [103] that the coating can effectively prevent corrosion and biofouling on metal surfaces, implying its great potential as a protective coating in practical engineering processes. In another study [120], a smart SH silicone-based coating has been developed by a disulfide exchange reaction of the functionalized monomer “lipoic acid-benzothiazole” (LA-BTZ) with LA-BTZ-modified PDMS-based polyurea-urethane. The obtained SHSM maintains good toughness (*σ* = 2.58 MPa), high stretchability (*ε* = 1000%), and SH properties. The authors noted that the adhesion strength of these silicone-based coatings to epoxy resin and steel surfaces was 2.5 and 3.3 MPa, respectively.

#### 3.1.2. Self-Healing Antifouling and Antimicrobial Coatings

Ongoing research [20,104,106,145] focuses on fabrication antibiofouling layers, which can coat and protect substrates from the growth of organisms such as bacteria, mold, mildew, or algae on substrates (e.g., marine structures). Thus, Sun J. et al. [104] fabricated a novel silicone-based poly(urea-thiourea)/tannic acid composite with excellent mechanical (*σ* = 2.47 MPa and high stretchability of 1000%), SH and antifouling properties. According to ref. [104], laboratory bioassays against marine bacteria adhesion (96, 95, and 93% reduction for *P. sp.*, *E. coli*, and *S. aureus*, respectively) and diatom attachment (84% reduction) showed an antifouling property of such SH films. In another ref. [106], the antifouling performance of polyurethane/fluorinated polysiloxane-microcapsules-silica was realized by constructing a micro–nano-dual-scale surface formed by the microcapsules and nano-SiO_2_.

In 2023, Wang P. et al. [20] prepared zwitterionic-functionalized metal-based PDMS antifouling coatings with SH properties. An interaction between vinyl-containing PDMS and triisopropylsilyl methacrylate-functionalized gallium-based liquid metal nanodroplets (TISM-GLM) with zwitterionic surfaces is the basis of the SH mechanism. According to the ref. [20], the well-dispersed TISM-GLM nanodroplets equip the PDMS coating with SH ability through the GLM nanodroplet-induced radical polymerization of vinyl groups in PDMS. The prepared films showed high antifouling efficacy inhibiting bacterial and algae adhesion (removing >96% of bacteria and >77% of algae).

In 2023, Guo R. et al. obtained a SH material based on *N*-acetyl-*L*-cysteine (NACL) [100]. This amino acid was grafted by hydrothyolation reaction to vinyl-containing PDMS obtained by a ring-opening anionic polymerization of 1,3,5,7-tetravinyl-1,3,5,7-tetramethylcyclotetrasiloxane (Figure 10i). Due to the presence of a hydrogen bond between the fragments of the carboxyl and amide groups in the amino acid, the *η* reaches 97% with a ratio of PDMS:NACL = 70:30. An interesting fact is the presence of antibacterial properties of the obtained material due to amino acid residues. As in a previous case [100], almost the same strategy was proposed [128] to prepare silicone elastomers with bio-based tannic acid as cross-linkers and 2,2-bis(hydroxymethyl)propionic acid as an intermediate chain extender. These materials exhibit not only an antimicrobial efficiency of over 90% and a final oxygen index of 26%, but also flame retardant properties [128].

Thus, similar antibacterial self–healing materials are very promising in the field of biomedicine [156] as, for example, antibacterial coatings and surfaces for operation hospitals, veterinary clinics, as well as the food industry [145].

#### 3.1.3. Self-Healing Anti-Icing Coatings

Anti-icing outdoor coatings definitely suffer from surface injuries such as extreme weathering, e.g., freezing weather or acid rain. The production of anti-icing SH coatings for extreme conditions is highly important. Thus, Li R. et al. [121] obtained an extreme-environment-resistant SH anti-icing coating by incorporating fluorinated graphene into a supramolecular polymeric matrix. The described coating can sustain its anti-icing/deicing performance after autonomous SH under harsh conditions, including low temperature (−20 °C), strong acid (pH = 0), and strong alkali (pH = 14) environments.

#### 3.1.4. Self-Healing Superhydrophobic Coatings

Special attention should be paid to obtaining superhydrophobic coatings [26,76,119,155] as well as to corrosion protection [117] and anti-icing [52]. Since superhydrophobic coatings are susceptible to stress due to the fragility of their structure, resulting in reduced superhydrophobic and anti-icing performance, in 2022–2023 some reports proposed a new insight to improve durability. It was implemented by introducing a thin layer of self-healable silicone elastomer with dynamic networks, based on reversible boroxine and hydrogen bonds [52,119,155]. In 2022, Kaili J. et al. [117] fabricated conductive and superhydrophobic silicone surfaces emerging a superior application potential in the outdoor equipment deicing field, due to their outstanding electrothermal heating capacity. The superhydrophobicity and conductivity kept unchanged when it was exposed to strong acid, alkali and salt, maintaining superhydrophobicity (151.5°) and conductivity (0.96 Ω⋅sq^−1^).

### 3.2. Recent Developments in Electromagnetic Interference Shielding Films

In the last few years, SH silicone composites began to be actively used to create electromagnetic shielding films and coatings [57,99], which prevent electromagnetic waves or radiation, protect electronic information from leakage, and resist electromagnetic interference. Thus, in 2021, a highly colorless, tear-resistant and compliant SHSM was developed by Sun F. et al. [99], which was tailored for transparent electromagnetic interference shielding films. The authors proposed a strategy to design a highly dynamic polyurea elastomer characterized by high optical transparency of >94%, ultralow elastic modulus (<1 MPa), high tear-resistant stretchability (*ε* = 800%), and ultrafast autonomous SH (100 s for scratch-healing). Taking the polymer as a substrate for embedding Ag nanowires, the first transparent, stretchable and self-healable electromagnetic interference shielding materials were prepared.

**Figure 18:**
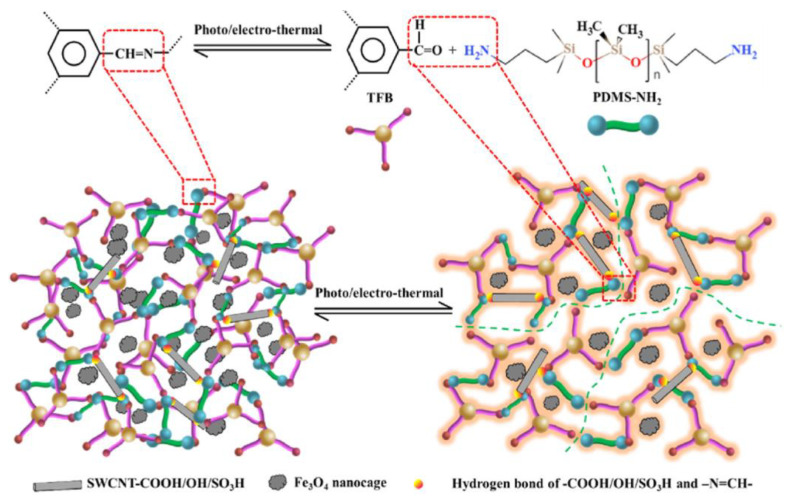
Structure and reaction process of the photothermal-thermoelectric composite film [57]. Copyright 2022, Elsevier.

A year later, Wu S. et al. [57] assembled an excellent photothermal-thermoelectric PDMS/single-walled carbon nanotube@Fe_3_O_4_ composite film, owning superior electromagnetic wave attenuation, SH and low temperature resistance. The SH caused by reversible imine and hydrogen bonds (Figure 18). The optimized composite film delivered a strong absorption and effective absorption of 3 GHz. The authors noticed that [57] the only indirect solar power generation electromagnetic wave absorption material by thermoelectric and photothermal effects with extreme environmental tolerance.

### 3.3. Recent Developments in Flexible Sensors

Recently, SHSMs have been used as sensors, especially strain sensors and wearable electronic sensors [49,61,101,118,124,129,130,131,134,136,141,143,147,148,157], pressure sensors [127,130], pH- and chemosensors [144,146].

#### 3.3.1. Flexible Strain Sensors

Strain sensors detect occurring stresses, and are referred to as strain transducers or a special form of force sensors. SH flexible and stretchable silicone rubbers can be utilized as components for flexible electrodes, which are usually offered for use as a strain sensors (“electronic skins”) to monitor human motions, including different bending angles of fingers and elbows [147,148]. Strain sensors with long-term durability, high sensitivity, and stretchability are required for the fabrication of flexible and wearable electronic devices. Therefore, SHSMs as ideal substrates are widely used in flexible electronics.

In 2021, Yu T. et al. [101] designed and prepared a SH elastomer, in which a quadruple hydrogen bonding network was constructed by introducing 2-ureido-4[*1H*]-pyrimidinones into polysiloxane. A three-layer strain sensor with high sensitivity and durability was fabricated using this twistable and highly stretchable (up to 2000%) silicone material as the substrate that was able to monitor various activities of the human body accurately. In 2022, Zhang T. et al. [49] used “hierarchical” covalent cross-linked networks and reversible disulfide covalent bonds to construct SH siloxane elastomers constructed for flexible electronics. Tang M. et al. [118] obtained ultrafast SH and self-adhesive polysiloxane towards reconfigurable on-skin electronics using dynamic hydrogen and boronic ester bonds as cross-links. The novel polysiloxane not only achieved a satisfactory tensile properties (*σ* = 0.43 MPa, *ε* = 1500%), but also recovered 100% of its original strength at RT within only 30 s after damage. In 2022, other robust SH elastomers [129] were created based on competing non-covalent interactions, and can be used as a flexible substrate to easily fabricate SH electrodes.

In 2023, SH, recyclable, mechanically tough transparent silicone elastomers based on dynamic microphase separation for flexible sensors were reported [143]. The elastomers include disulfide and hydrogen bonds, which enable strong microphase separation, providing this series of SHSMs with the *σ* = 1.89–3.33 MPa, *ε* = 350–1720%, and an extreme fracture toughness of 28.6 MJ·m^−3^. A “sandwich-structure” flexible sensor device, which can be cut and heal just like building blocks, was designed that can be utilized for detecting human motions.

**Figure 19:**
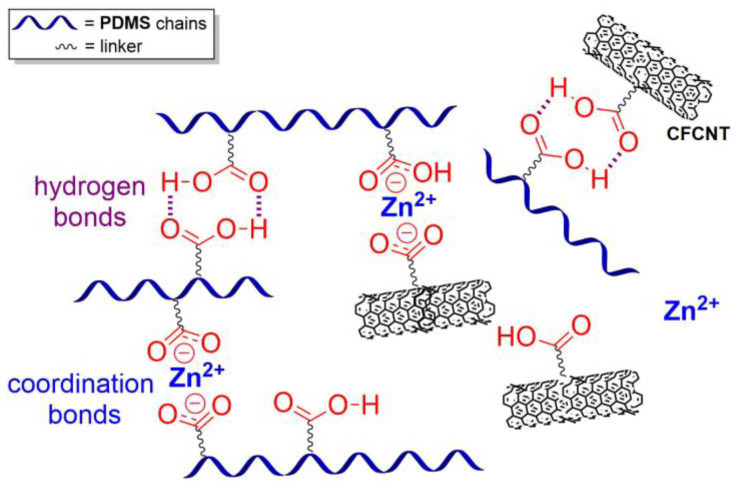
Structure of self-healing conductive elastomers [131].

In order to fabricate strain sensors, the conductivity of polysiloxanes should be increased by the preparation of nanocomposites with conductive fillers, especially multi-walled carbon nanotubes (MWCNT), carboxyl-functionalized carbon nanotubes (CFCNT), AgNPs, etc. Thus, in 2021, Mail D. et al. developed [134] a bilayer SH strain sensor consisting of CFCNT and ionically cross-linked polysiloxane substrates, based on unsaturated acid−amine interactions. The sensor exhibited self-adhesiveness, high sensitivity, linearity, low hysteresis, and long-term durability with a gauge factor of 34 at 55% strain. In the same time, SH PDMS/AgNPs conductive elastomer with tunable tensile properties and efficient antibacterial performances for a wearable sensor was developed (Figure 14f) [141]. The highly stretchable elastomer (*ε* = 1760%) can accurately monitor the bending motion of human joints. In 2022, Zhang K. et al. [131] developed hyperbranched-MWCNT/hyperbranched-PDMS self-healable conductive elastomers inspired by cephalopods, which were sensitive to the change of stress states and can be used as a stable strain sensor. The prepared conductive elastomer exhibited autonomous SH properties (*η* > 90%) at RT, excited by multiple reversible interactions (coordination and hydrogen bonds) (Figure 19). The prepared elastomer also showed excellent anti-fatigue ability.

A high electrical SH flexible strain sensor (ESFSS) based on a nanocomposite of MWCNT and PDMS elastomer (comprising reversible imine and hydrogen bonds) was also proposed by Yu T. et al. [124]. The conductive fillers were incorporated into the sensor in order to endow the ESFSS with high sensitivity and a wide measurement range. The high electrical SH performance of the ESFSS was shown with an electrical *η* = 98%. The proposed ESFSS also has high gauge factor values of 58 (0–85%), 993 (85–107%) and 5536 (107–136%), and a wide measurement range (0–136%).

#### 3.3.2. Flexible Pressure Sensors

The creation of piezoresistive pressure sensors with SH ability has attracted much attention, due to their potential applications in intelligent and soft robots, remote health monitoring, and electronic skins [127,130]. Thus, Yang Z. et al. [127] designed a conductive SH silicone (CSE) film with a ridge-like microstructure for flexible piezoresistive pressure sensors. In order to obtain a CSE film, the PDMS-based elastomer with imine and ureido groups was synthesized by a polycondensation of aminopropyl-terminated PDMS with isophorone diisocyanate and 1,3,5-triformylbenzene, the solution of which was cast onto a sandpaper pre-sprayed with ureido pyrimidinone-grafted carbon nanotubes/polyurea mixed solution and dried. The piezoresistive pressure sensor was fabricated with two single-electrode CSE films maintained a high sensitivity of 8.7 kPa^−1^ (0–6.1 kPa), low detection limit (50 Pa), fast response capability (response/relaxation time of 40/117 ms), and repeatability for 10,000 loading–unloading tests. The authors reported [127] that this pressure sensor was applied not only to detect human motions (radial pulse, voice recognitions and joint movements), but also to monitor health status wirelessly through Bluetooth transmission.

### 3.4. Recent Developments in Actuators

Electroactive dielectric elastomers demonstrate great potential for a variety of miniature transducers, due to their unique mechanical and electromechanical properties (i.e., high strain and energy density, fast response speed, and inherent environmental tolerance) [158]. Silicone rubbers satisfy these requirements [8,44,66,107,118,126,147,148,150]. One of the first and most notable results on the creation of actuators based on autonomous PMCs of poly(2,6-pyridinedicarboxamide-*co*-PDMS) coordinated with Fe(III) was obtained by Bao Z. et al. [66] in 2016. The ability of this SHSM to restore a high dielectric strength after healing from mechanical damage, as well as ultrahigh stretchability (up to 10,000%), provides its promising artificial muscle applications.

In 2022, Feng Z. et al. [44] reported a thermo-reversible furfuryl poly(thioether)-*b*-polysiloxane-*b*-furfuryl poly(thioether) triblock copolymer with tunable dielectric and mechanical performances. The resultant material maintains a mechanical strength of 0.16–11.2 MPa), a high dielectric permittivity, self-heals by Diels–Alder bonds, and possesses outstanding shape memory and shape reconfiguration behavior with a fixing ratio and recovery ratio of >90%. According to the authors’ opinion, preparation of such a homogenous dielectric elastomer with improved electromechanical performance and shape memory behavior leads to promising application prospects in actuated devices.

**Figure 20:**
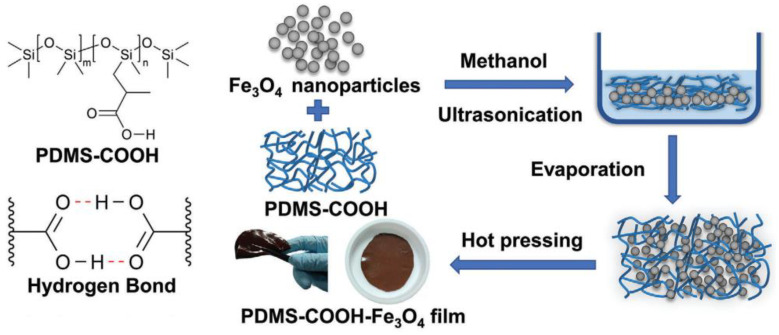
Schematic diagram of the preparation of magnetic nanocomposite [107]. Copyright 2021, John Wiley and Sons.

In the same year, Tao H.-T. [107] used magnetic effects for actuation by obtaining autonomous SH magnetic nanocomposite using an environmentally friendly strategy. The nanocomposite comprised a soft PDMS as the polymer matrix and Fe_3_O_4_ nanoparticles as functional magnetic nanofillers in an optimal concentration of 15 wt.% (Figure 20). The most optimized sample possessed *σ* = 0.44 MPa, a high *ε* = 400%, and *η* = 62% after healing at 25 °C for 30 min. The nanocomposite material exhibits a healable magnetic actuation performance, providing great potential for the magnetic actuation applications.

Zang W. et al. [111] developed a dielectric elastomer generator (DEG), which is a kind of deformable elastic capacitor composed of a thin dielectric elastomer film sandwiched between two compliant electrodes. A DEG can convert the electrical energy into mechanical energy under electrical excitation. According the ref. [111], the prepared conductive rubber electrode had high electrical conductivity, high strain and high durability during cyclic stretching, SH and recycling ability. The conductive rubber comprises a hydrogen-bond-cross-linked network of silicone rubber and highly conductive carbon black and carbon grease. Meanwhile, this electrode demonstrates high *η* of conductivity (92% after healing at 60 °C for 4 h), and can be recycled five times without negative effects on performance. Thus, this DEG can be used as dielectric elastomer actuator that finds application in artificial muscles, etc.

In 2023, Szczepanski J. [8] prepared a SH silicone elastomer with a large and tunable permittivity by an anionic ring-opening copolymerization of cyanopropyl-substituted cyclic and tricyclic (*tris*-D_4_) oligosiloxanes (Figure 2d). The silanolate end groups remain active after preparation of the materials that cause SH by siloxane equilibrium. According to the ref. [8], the high elasticity of the materials is essential for reversible actuation, and the thermoreversible softening allows for SH and recycling. Fabricated single-layer actuators on their basis showed 3.8% lateral actuation at 5.2 V·μm^−1^ and SH after an electrical breakdown. Stack actuators reached an actuation strain of 5.4 ± 0.2% at electric fields of 3.2 V·μm^−1^, and thereby providing their applications as artificial muscles in soft robotics.

### 3.5. Recent Developments in Triboelectric Nanogenerators

In 2021–2022, along with the actuators, some triboelectric nanogenerators based on SHSMs were fabricated [126,130]. They are an emerging powerful technology that converts ambient mechanical energy into electrical energy via a triboelectric effect. These devices are very promising in the field of electronic skin. Thus, Jiang J. et al. [130] created an ultrastretchable triboelectric nanogenerator that simultaneously heals fractures and abrasions at RT (100% efficiency), as well as exhibits ultrahigh stretchability (up to 10,000%). The SH in this material occurs due to the incorporating of hydrogen bonds and dynamic metal-ligand coordination into PDMS chains (Figure 14d). Working in contact-separation mode, the electrical outputs with a 2 × 2 cm^2^ area can reach 140 V, 40 nC, and 1.5 µA, respectively. According to the ref. [130], if the nanogenerator is stretched to break or scratched to wear out, it can restore its electrical outputs in 20 min and 2 h at RT. Cai Y.-W. et al. [126] also reported a nanogenerator consisted of a triboelectric layer (SH double-cross-linked PDMS obtained by adjusting the ratio of imine bonds to hydrogen bonds) and an electrode layer (SH conductive composite). This triboelectric nanogenerator exhibits shape adaptability, and thereby can be perfectly attached on an uneven human skin surface, and can maintain the original triboelectric performance after repeated damage.

Consequently, the application of the described nanogenerators as flexible power sources and self-powered pressure sensors was also demonstrated, leading to the broad applications of flexible and wearable electronics for long-term use [126,130].

### 3.6. Recent Developments in Luminescent and Electroluminescent Devices

The inclusion of lanthanides and other luminescent ions, as well as conjugated and flat aromatic fragments, into the structure of SHSMs can give them an additional property of photoluminescence, which contributes to the expansion of potential applications of such materials, and their use in optoelectronics as flexible SH screens, fluorescent mosaics, lighting design, etc. [18].

**Figure 21:**
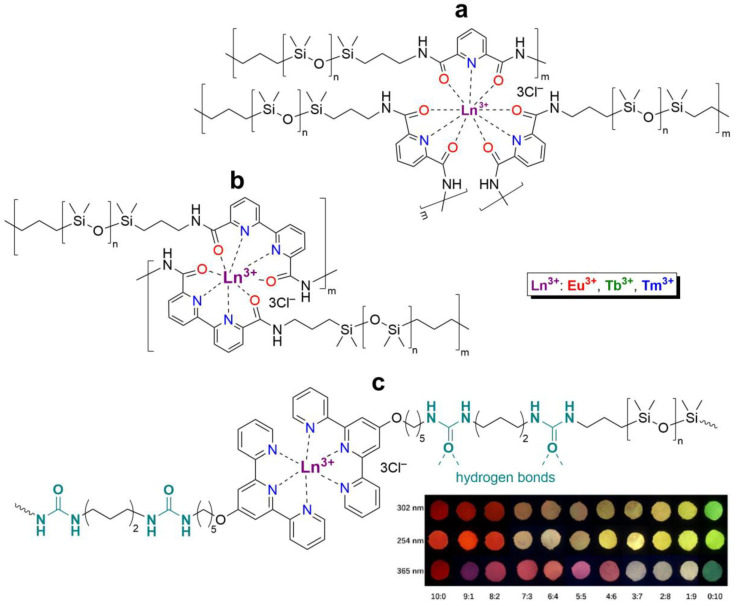
The proposed structures of lanthanide-containing PMC with 2,6-pyridinedicarboxamide (**a**) [86], bipyridinic (**b**) [65], and terpyridinic fragments with optical images excited under 302, 254, and 365 nm (from right to left Eu^3+^:Tb^3+^ = 10:0, 9:1, 8:2, 7:3, 6:4, 5:5, 4:6, 3:7, 2:8, 1:9, 0:10) [132] (**c**). Copyright 2022, Elsevier.

The first type of photoluminescent SH silicone rubbers are polymer-metal complexes (PMC) of PDMS-containing polymer ligands and lanthanide ions, especially Eu^3+^ and Tb^3+^ [65,80,86,132,146,159]. The presence of lanthanide ions in the structure of such polymers causes their phosphorescence, and contributes to a sufficiently high photoluminescence quantum yield. Since characteristic *4f–4f* electronic transitions have a forbidden nature, the lanthanide ions have a long photoluminescence lifetime and sharp spectral lines. Thus, to increase photoluminescent quantum yield (up to 30 and 40% for Eu- and Tb-PMC, respectively, the “antenna effect” should be achieved by using sensitizer ligands including 2,6-pyridinedicarboxamide [86], bipyridinic fragments [65], terpyridinic fragments [132], or *β*-diketone [146] (Figure 21).

The photoluminescence color can be controlled (*i*) by the Eu^3+^:Tb^3+^ ratio, leading to obtaining a SHSM with an emission color close to white light, that can be applied as a phosphor for white light-emitting diodes [86], (*ii*) by making monolithic “sandwiches” using non-autonomous SH at 100 °C [65], and (*iii*) by choosing the excitation wavelength [132].

Kim E. et al. [146] synthesized cross-linked luminescent polymers based on Eu^3+^ and multiligand PDMS with grafted *β*-diketone fragments, which maintain tunable optical, mechanical properties, and chemosensory properties to ammonia cation.

**Figure 22:**
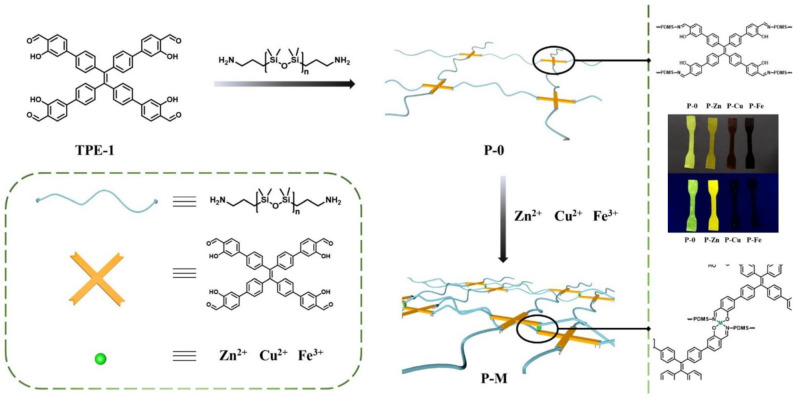
The proposed 3D polymer networks’ structure of SH tetraphenylene-containing PDMS cross-linked with various metal cations and their fluorescent properties [83]. Copyright 2023, Royal Society of Chemistry.

The second type of photoluminescent SH silicone rubbers contain flat conjugated aromatic fragments in a 3D polymer network [56,83]. In 2022, Wang N. et al. [56] obtained two aldehyde-modified tetraphenylene derivatives, and incorporate them into PDMS networks through reversible imine cross-linking. The prepared elastomers showed fluorescence properties, sufficient mechanical characteristics, thermal stability, and SH and recycle properties. In this case, the SH process takes place quickly, and the recycling process can be carried out by solution processing and hot pressing. In 2023, the same scientific group [83] prepared SHSMs with excellent tunable mechanical and fluorescence properties based on structures with dual cross-linking through reversible covalent imine and metal coordination bonds. Thus, salicylaldehyde-modified tetraphenylene derivatives with aggregation-induced emission properties were included into the dual cross-linked network as cross-linking sites (Figure 22). The mechanical properties and fluorescence of the considered SHSMs can be tuned by adjusting the type and content of metal ions.

SHSMs can be used not only to create flexible luminescent devices, but also self-powered multi-color displays based on stretchable alternating current electroluminescent (ACEL) devices [55] with the triboelectric nanogenerators. The obtained material has superb stretchability (2500%) and a high *η* (96%) at RT, due to the reversible dynamic imine bonds. The ACEL devices with such SH and stretchable PDMS as the substrate of electrodes and the matrix of emission layers were constructed.

### 3.7. Recent Developments in Solar Cells

**Figure 23:**
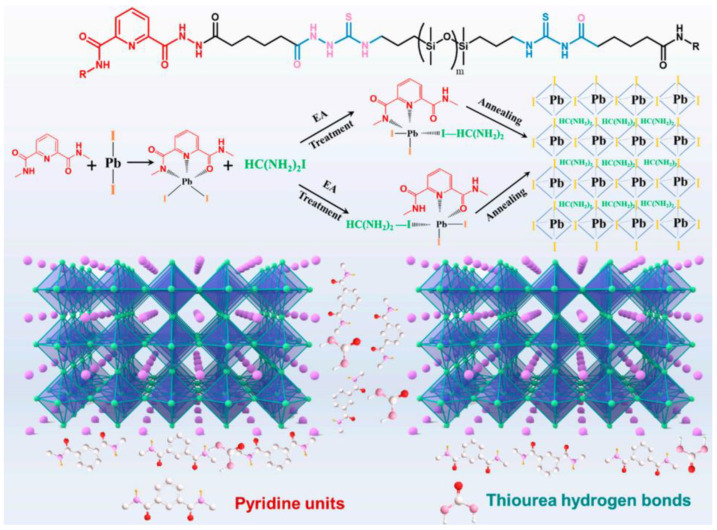
Proposed mechanisms of passivation and SH characteristics, and the physical structure of devices [138]. Reproduced with permission from *Nanomaterials*; published by MDPI, 2022.

If perovskites are introduced into the structure of SHSMs by the interaction between perovskite nanoparticles and polysiloxane functional groups, then it is possible to obtain a material suitable for use as solar cells [137,138]. In 2022, Zhang K. et al. [137,138] designed a brand-new SH polysiloxane with dynamic 2,6-pyridinedicarboxamide (PDCA) coordination units and plenty of hydrogen bonds, and incorporated it into perovskite films. According to the ref. [137], PDCA units, showing strong intermolecular Pb^2+^–N_amido_, I^−^–N_pyridyl_, and Pb^2+^–O_amido_ coordination interactions, were expected to enhance crystallinity and passivate the grain boundary (Figure 23). Reversible urea and thiourea hydrogen bonds in a 3D silicone network afforded the SH of cracks at grain boundaries for fatigue perovskite solar cells. This strategy of doping in perovskite solar cells opens up an opportunity to realize efficient and durable crystalline semiconductors [137,138]. In summary, polymer doping is an important approach to improve the electronic quality of perovskite solar cells fabricated in air.

In 2022, Sun J. et al. [113] fabricated a SH film for solar-thermal applications by employing a diradical-featured organic small-molecule croconium derivative (CR-DPA-T) as a solar harvester, loaded flexible SH H-PDMS film. The autonomous SH caused by π–π-stacking of CR-DPA-T into dimer form.

## 4. Conclusions

Thus, great steps in the development of SHSMs have been taken over the past few years. If earlier, about 10–20 years ago, only the SH mechanisms in dynamic 3D polymer networks were studied, then over the past five years, emphasis has been placed on the combination of several types of reversible bonds into polysiloxanes, and the use of such silicone materials in high-tech and significant areas of materials science.

The reversible chemical interactions used in SHSMs are classified as covalent and non-covalent bonds. SHSMs based on covalent interactions exhibit predominantly non-autonomous SH properties and require external action (heating, UV, the additional reaction agent), which is associated with stronger bonds in their structure and increased dissociation energy. This cannot be called an unambiguous advantage or disadvantage since, in some cases, SHSMs can be applied if they self-heal only under special conditions.

One of the important features of the metal-ligand coordination bonds, which belongs to the category of covalent interactions, is that the mechanical and SH material’s properties can be relatively easily controlled by changing the structure and molecular weight of the polysiloxane ligand, metal–polysiloxane ligand ratio, and metal ion and counterion content.

Non-covalent bonds are weaker interactions compared to covalent ones, that mainly lead to autonomous SH (especially hydrogen and ionic bonds). However, it is necessary to form a sufficiently large number of weak non-covalent bonds to form stable, mechanically strong, and durable SH polymer structures.

Along with the huge benefits of self-healing materials [3], side effects can often lead to many of these SHSMs being sticky and able to dissolve (completely or partially) in contact with some solvents [19,50,51,64], which can lead to some issues for their potential applications. In this regard, SHSMs with combined interactions and nanofillers are being developed to solve the drawbacks mentioned before. Nanocomposites with a certain range of properties (electrical conductivity, photoluminescence, electroluminescence, magnetic properties, high mechanical strength, durability, etc.) are very often created based on SHSMs with covalent (Diels–Alder reactions, coordination bonds, etc.) and non-covalent type of reversible bonds (hydrogen bonds, etc.) between NPs and polysiloxane matrix in their structure.

SHSMs can possess not only typical SH characteristics, but also some additional properties, which are useful for their extension of application, including thermal and cold-resistance, redox-activity, photoluminescence, high dielectric properties, electrical conductivity, antifouling, antimicrobial, anti-icing properties, etc.

The application fields of SHSMs has expanded significantly. For instance, SH silicone rubbers maintain broad applications in nanotechnology, optoelectronics, biomedicine, additive manufacturing, soft robotics and human activity as self-repairing protective coatings, electromagnetic shielding films, sensors and skin-inspired electronics, actuators and artificial muscles, triboelectric nanogenerators, flexible or stretchable luminescent and electroluminescent devices, and solar cells.

However, the application potential of SHSMs has not been fully disclosed. In this regard, the development of this field of polymer chemistry and materials science poses challenging tasks for the scientific community.

## Data Availability

All data reported herein accompany the present article.
